# Mass spectrometry-based analysis of cerebrospinal fluid from arthritis patients—immune-related candidate proteins affected by TNF blocking treatment

**DOI:** 10.1186/s13075-019-1846-6

**Published:** 2019-02-15

**Authors:** Johanna Estelius, Johan Lengqvist, Elena Ossipova, Helena Idborg, Erwan Le Maître, Magnus L. A. Andersson, Lou Brundin, Mohsen Khademi, Elisabet Svenungsson, Per-Johan Jakobsson, Jon Lampa

**Affiliations:** 1 Rheumatology Unit, Department of Medicine, Solna, Center of Molecular Medicine (CMM), Karolinska Institutet, Karolinska University Hospital, SE-17176 Stockholm, Sweden; 2 Neuroimmunology Unit, Department of Clinical Neuroscience, Center of Molecular Medicine (CMM), Karolinska Institutet, Karolinska University Hospital, SE-17176 Stockholm, Sweden

**Keywords:** Chronic inflammation, CSF, Intrathecal inflammation, Anti-TNF, Proteomics, Ankylosing spondylitis, Psoriatic arthritis, Juvenile chronic arthritis, Rheumatoid arthritis

## Abstract

**Background:**

Signs of inflammation in cerebrospinal fluid (CSF) of rheumatoid arthritis patients correlate positively with fatigue, a central nervous system (CNS)-related symptom that can be partially suppressed by TNF blockade. This suggests a possible role for CNS inflammation in arthritis that may be affected by TNF blockade. We therefore investigated the effects of TNF blockade on the arthritis CSF proteome and how candidate proteins related to clinical measures of disease activity and inflammation.

**Methods:**

Mass spectrometry-based quantitative proteomic analysis was performed on CSF from seven polyarthritis patients before and during infliximab treatment. Treatment-associated proteins were identified using univariate (Wilcoxon signed rank test) and multivariate (partial least squares discriminant analysis (PLS-DA)) strategies. Relations between selected candidate proteins and clinical measures were investigated using the Spearman correlations. Additionally, selected proteins were cross-referenced to other studies investigating human CSF in a thorough literature search to ensure feasibility of our results.

**Results:**

Univariate analysis of arthritis CSF proteome revealed a decrease of 35 proteins, predominantly involved in inflammatory processes, following TNF blockade. Seven candidate proteins, Contactin-1 (CNTN1), fibrinogen gamma chain (FGG), hemopexin (HPX), cell adhesion molecule-3 (CADM3), alpha-1B-glycoprotein (A1BG), complement factor B (CFB), and beta-2-microglobulin (B2M), were selected for further studies based on identification by both univariate and multivariate analyses and reported detection in human CSF and known associations to arthritis. Decreased levels of FGG and CFB in CSF after treatment showed strong correlations with both erythrocyte sedimentation rate and disability scores, while CNTN1 and CADM3 were associated with pain.

**Conclusion:**

Several immune-related proteins in the CSF of arthritis patients decreased during TNF blockade, including FGG and CFB that both correlated strongly with systemic inflammation. Our findings stress that also intrathecal inflammatory pathways are related to arthritis symptoms and may be affected by TNF blockade.

**Electronic supplementary material:**

The online version of this article (10.1186/s13075-019-1846-6) contains supplementary material, which is available to authorized users.

## Background

Central nervous system (CNS)-related symptoms such as pain, fatigue and cognitive dysfunction are common features of several chronic inflammatory disorders [1, 2] and may persist despite achieving good control of peripheral inflammation [3]. We have previously detected elevated inflammatory mediators in cerebrospinal fluid (CSF) of rheumatoid arthritis (RA) patients, where increased interleukin-1beta (IL-1β) levels correlated positively with fatigue [4]. Moreover, arthritis is associated with disturbed central pain regulation as reviewed by Walsh et.al. [5]. Together, this indicates a connection between arthritis, central nervous mechanisms and global CNS symptoms.

Tumour necrosis factor alpha (TNFα) has been linked to pain mechanisms as well as cognitive dysfunction [6, 7], and TNF-blocking treatment, e.g. infliximab, is described to ameliorate pain as well as fatigue in arthritis patients [8, 9]. TNF-blockade is an effective treatment with well-documented anti-inflammatory effects for several chronic inflammatory diseases, including different types of arthritis [10–13]. Interestingly, infliximab treatment has been shown to reduce levels of the pro-inflammatory cytokine interleukin-6 (IL-6), in both serum of RA patients [14] and serum and CSF of neuro-Behcet’s patients [15, 16] indicating possible effects on neuro-inflammation. Additionally, animal studies of TNF blockade indicate considerable benefits on CNS and CNS-related symptoms [6, 17, 18]. However, little else is known about the effects of TNF blockade in the human intrathecal compartment or the relationship between CSF effects, CNS-related arthritis symptoms and peripheral inflammation.

Proteomic analysis of CSF as an approach to derive biomarkers for neurological and inflammatory events has been pursued over the last decade [19, 20]. However, few proteomic studies of CSF directly relate to CNS-related symptoms [21]. Using proteomic profiling approaches thus enables identification of novel biomarker candidates.

In the current work, we investigate the effects of TNF blockade (infliximab) on CSF protein levels in patients with polyarthritis, and candidate protein relationships to clinical measurements of disease activity, peripheral inflammation, function and patient-reported outcomes.

## Methods

### Patients and controls

CSF was obtained by lumbar puncture from ten female polyarthritis patients attending the Rheumatology clinic at Karolinska University Hospital, Stockholm, Sweden. Samples were collected before (baseline) and after 8 weeks of infliximab treatment. For three patients, CSF sample was collected from only one occasion. These three patients were excluded from consecutive analyses. Control CSF samples were acquired from ten age- and sex-matched patients (median age 42 years, range 27–72 years) with non-inflammatory neurological diseases (NINDC) including psychosis (*n* = 3), vertigo (*n* = 2), migraine (*n* = 1), tension headache (*n* = 1), paresthesia (*n* = 1), paraparesis (*n* = 1) and trigeminal neuralgia (*n* = 1), undergoing diagnostic workup at the Neurology Clinic at Karolinska University Hospital, Stockholm, Sweden. All patients gave their informed written consent. The study was approved by the local ethics committee at Karolinska University Hospital and complies with the declaration of Helsinki.

### Treatment schedule and CSF handling and quality control

A standard treatment schedule was followed with intravenous infliximab given at weeks 0, 2 and 6. Standard doses of 3 mg/kg were used. In one patient, the treatment was temporarily withdrawn after the second infliximab infusion because of a knee arthroplasty. In this patient, infliximab was given at weeks 0, 2 and 14, with sampling at baseline and after week 14.

Acquired CSF samples were immediately centrifuged, and the pellet and supernatants were recovered and stored in − 70 °C until used as described previously [22, 23].

Acquired CSF samples were visually inspected for blood contamination, and all samples were found to be clear and uncoloured. An additional erythrocyte count was performed on an aliquot taken from each sample prior to centrifugation. Seven out of nine baseline samples contained zero erythrocytes/μl while two baseline samples contained one and 13 erythrocytes respectively. For the follow-up samples, 8 out of 9 samples contained zero erythrocytes/μl while one sample contained 341 erythrocytes/μl. The sample containing 341 erythrocytes/μl belonged to a patient without paired CSF samples and was not included in further analysis.

### Clinical assessments

#### Disease activity, pain and health assessment

Disease Activity Score 28 (DAS28) is a composite measure of the number of tender and swollen joints in 28 locations, patient-estimated global health on a 100-mm visual analogue scale (VAS patient global health) and erythrocyte sedimentation rate (ESR) [24]. It was used to assess disease activity in patients at baseline and week 8 of CSF sampling. Using a 100-mm visual analogue scale (VAS-pain) ranging from “no pain” to “worst imaginable pain”, patients were asked to rate their overall perception of pain [25].

Health assessment questionnaire (HAQ) is a questionnaire addressing patient-reported outcomes, in its full form assessing five dimensions: disability, pain, medication effects, cost of care and mortality [26]. In this study, the short form HAQ only addressing disability is used at each sampling occasion.

### Proteomic profiling

#### Sample preparation

Protein concentration of CSF samples was determined using NanoDrop™ 2000 Spectrophotometer (Thermo Fisher Scientific). Aliquots of 50 μg total protein of CSF samples were mixed with 50 μl of 0.05 M triethyl ammonium bicarbonate (TEAB) buffer, reduced by 0.1 M tris(2-carboxyethyl) phosphine hydrochloride (TCEP) for 1 h at 37 °C and alkylated using 0.5 M iodoacetamide for 45 min at RT in darkness. Peptides were obtained by digestion using trypsin (1.50 enzyme:substrate) at 37 °C overnight (ON). Peptides were desalted by Pierce™ C18 Tips according to the manufacturer’s instructions and dried in speed-vac prior LC-MS/MS analysis.

#### LC-MS/MS analysis

Analysis of the peptide mixtures was performed by Dionex HPLC system (Thermo Scientific) equipped with Acclaim PepMap™ RSLC (75 μm × 50 cm) column coupled to the Bruker CaptiveSpray electrospray source installed onto the Bruker Daltonics impact II™ oQTOF instrument. Each acquired spectrum was calibrated internally using sodium formate cluster ions by injection of the calibration mixture in the beginning of LC gradient.

#### Protein identification and quantification

Acquired data were analysed against human protein sequences from Uniprot (downloaded 2015.01.17) using Mascot search engine (Matrix Science) incorporated into the ProteinScape™ platform (Bruker). In Mascot searches, tolerance of 10 ppm for precursor masses and 0.2 Da for fragment ions were specified. Searches were performed with trypsin specificity, two missed cleavages were allowed, and carbamidomethylation was set as static modification and oxidised methionine as dynamic modification. Identified proteins were filtered to a 1% FDR cut-off.

Normalised spectral abundance factor (NSAF) was calculated on the basis of peptide spectral counts for each protein detected in more than two arthritis samples. NSAF values were used in further analyses.

### Statistical analysis

For univariate analysis of proteomic data, comparisons between control (NINDC) and arthritis groups were made by Mann Whitney *U* test and between paired arthritis samples by Wilcoxon signed rank test. Associations to clinical parameters were calculated using the Spearman correlations. All of the univariate data analyses were calculated using SPSS v. 23 (IBM, Armonk, NY, USA) with significance level set at *p* < 0.05. Data are presented as median (interquartile range) unless stated otherwise.

Multivariate data analyses (principal component analysis (PCA) and partial least squares discriminant analysis (PLS-DA)) were performed using SIMCA P+ version 12 (MKS Data Analytics Solution, Umeå, Sweden). Prior to analysis, the data was mean centred and scaled to unit variance. Proteins important for the separation of patient CSF samples before and after treatment using the PLS-DA model species were selected based on a combination of variable influence in projection (VIP ≥ 1.5) and scaled loadings (*p* (corr) ≥ |0.5|).

## Results

### Short-term effects of infliximab treatment on systemic inflammation and clinical measures of function, pain and disease activity in polyarthritis patients

Ten polyarthritis patients were here followed during the first 8 weeks of infliximab treatment. At two sampling occasions (baseline and after 8 weeks of infliximab treatment), CSF samples as well as information on peripheral inflammatory status (C-reactive protein (CRP) and erythrocyte sedimentation rate (ESR) levels), disease activity score 28 (DAS28), visual analogue scale pain (VAS-pain) and functioning (health assessment questionnaire (HAQ)) were acquired. On a group level, disease activity was decreased during the infliximab treatment (DAS28, 5.1 (3.9–6.2) BL vs. 4.4 (3.2–5.7) IFX, *Z* = − 2.03, *p* = 0.042). Comparing baseline (BL) and week 8 (IFX) measures in all samples, significant treatment effects were observed also on systemic inflammatory parameters (CRP, 19.8 (5.1–62.1) mg/L BL vs. 1.7 (0.7–6.2) mg/L IFX, *Z* = − 2.67, *p* = 0.008, and ESR, 35.5 (16.3–90.0) BL vs. 19.5 (11.3–43.3) IFX, *Z* = − 2.10, *p* = 0.036) as well as clinical assessments (VAS-pain, 75 (57–80) mm BL vs. 22 (6–75) mm IFX, *Z* = − 2.03, *p* = 0.043; HAQ 1.5 (0.9–1.7) BL vs. 0.9 (0.8–1.5) IFX, *Z* = − 2.20, *p* = 0.028). Patient characteristics are described in Table 1.

**Table 1:** Demography and clinical characteristics of polyarthritis patients (baseline and after 8 weeks of infliximab treatment)

ID	Age	Sex	Diagnosis	Additional treatments	Baseline					After infliximab treatment				
					DAS28	ESR (mm)	CRP (mg/L)	TJC	SJC	DAS28	ESR (mm)	CRP (mg/L)	TJC	SJC
1	43	F	RA^+^	M, N, Pa	3.80	18	5.4	1	1	3.82	15	ND	1	0
2	34	F	RA^−^	M, N, Pa	4.77	18	3.0	4	4	4.39	29	2.3	4	0
3	74	F	RA^−^	M, Pr, Pa	7.50	90	93.3	13	12	6.67	50	43.3	13	11
4	35	F	JCA	M, Pr, N	4.10	11	7.6	7	4	2.96	10	7.4	4	0
5	41	F	JCA	M, Pr, Pa	2.35	10	4.0	0	0	ND	ND	0.5	ND	ND
6	26	F	AS	M, Pr, N	ND	90	56.5	ND	ND	ND	24	1.7	ND	ND
7	52	F	PsA	M, N	6.34	90	78.8	6	4	4.73	48	ND	4	2
Patients without paired CSF samples														
8	31	F	RA^+^	M, N, Pa	ND	41	19.8	15	15	ND	12	0.5	ND	ND
9	51	F	RA^+^	M, A	5.94	30	15.6	12	3	ND	11	1.6	1	2
10	61	F	PsA	M, N, Pa	5.51	63	28.6	2	4	ND	ND	0.9	0	0

*A* azathioprine, *AS* ankylosing spondylitis, *CRP* high-sensitivity C-reactive protein, *DAS28* Disease Activity Score 28, *ESR* erythrocyte sedimentation rate, *F* female, *JCA* juvenile chronic arthritis, *M* methotrexate, *N* non-steroidal anti-inflammatory drug, *ND* not determined, *Pa* paracetamol, *Pr* prednisolone, *PsA* psoriatic arthritis, *RA*^−^ rheumatoid factor negative rheumatoid arthritis, *RA*^+^ rheumatoid factor positive rheumatoid arthritis, *SJC* swollen joint count, *TJC* tender joint count

### Short-term infliximab-induced effects on the CSF proteome of arthritis patients identified by proteomic profiling

Intrathecal effects of TNF-blockade on CSF proteome in patients with polyarthritis at the baseline and after 8 weeks of infliximab treatment (*n* = 7) were investigated. Normalised spectral abundance factor (NSAF) was calculated for the 306 proteins that were quantified. A full list of the identified proteins and their statistical results are provided in Additional file 1: Table S2. Univariate Wilcoxon signed rank test revealed that intrathecal levels of 31 of the 306 identified proteins were significantly altered after infliximab treatment (*p* < 0.05) (Additional file 1: Table S1). Among the 31 significantly altered proteins, several are involved in inflammatory processes including proteins belonging to the complement and coagulation systems. Interestingly, none of all the significantly changed proteins were increased; instead, all were reduced following infliximab treatment.

**Fig. 1:**
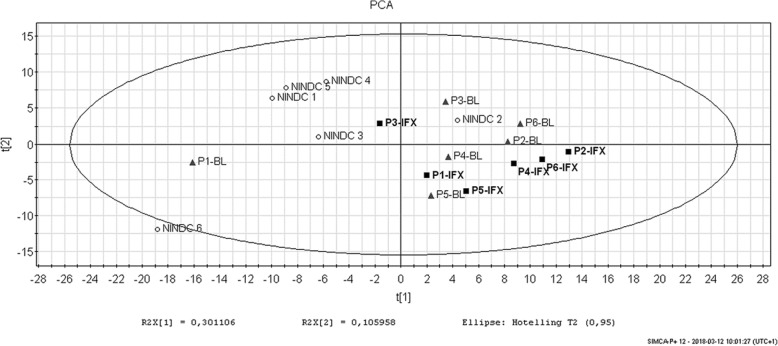
PCA score plot. A score plot based on PCA of label-free proteomics data including NSAF values of all detected proteins (*n* = 307) from paired CSF samples. Samples from polyarthritis patients at baseline (P-BL, grey triangles) and after 8 weeks of infliximab treatment (P-IFX, black squares) are shown as well as matched NINDC control CSF samples (open circles). MS data for one patient were excluded due to very high values of all detected proteins (pat-ID no 7)

Next, we performed a multivariate data analysis (principal component analysis (PCA)) on all proteins quantified by proteomics (*n* = 306). This analysis aimed to identify potential clustering of the patient samples at baseline and after infliximab treatment as well as clustering of arthritis and control (NINDC) samples. In our PCA model, 41% of the variance is explained by PC1 (30%) and PC2 (11%). As shown in Fig. 1, there are indications of clustering, with more pronounced separation between arthritis and NINDC control samples than between arthritis samples at baseline and after infliximab treatment. To focus on the difference between baseline and infliximab-treated proteomic profiles, a supervised partial least squares discriminant analysis (PLS-DA) model (*R*^2^*Y*_cum, 2PC_ = 0.98, *R*^2^*X*_cum, 2PC_ = 0.42, *Q*^2^*Y*_cum, 2PC_ = 0.62; CV-ANOVA > 0.05) was used to identify the proteins contributing most to the separation between arthritis CSF samples at baseline and after infliximab treatment (Additional file 2: Figure S1). Proteins selected (*n* = 27) (Additional file 1: Table S3) give the highest contribution to the potential difference between arthritis CSF samples before and after TNF blockade.

Of these 27 proteins, the following 11were also identified as significantly altered in CSF of arthritis patients after infliximab treatment by univariate analysis of the label-free proteomics data: cell adhesion molecule 3 (CADM3), Insulin-like Growth Factor-Binding Protein 7 (IGFBP7), Protein Tyrosine Phosphatase Receptor Type N (PTPRN), Apolipoprotein H (APOH), Alpha-1-B Glycoprotein (A1BG), Fibrinogen gamma chain (FGG), Beta-2-microglobulin (B2M), Complement C4B(C4B), Complement C7 (C7), Complement Factor B (CFB) and hemopexin (HPX) (Table 2).

**Table 2:** Proteins important for separation between patients before and after infliximab treatment

Protein name	Gene symbol	Accession	Univariate analysis			Multivariate analysis		Process	Suggested function(s)
			Fold change^†^	*Z* score	*p* value	VIP	*p* (corr)		
Cell Adhesion Molecule 3	CADM3	Q8N126	− 0.68	− 1.992	0.046	2.0	0.7	Cell adhesion	Overexpressed in murine microglia after bacterial challenge and may be involved in development of depressive symptoms following immune challenge. [43]
Insulin-like Growth Factor-Binding Protein 7	IGFBP7	Q16270	− 0.50	− 2.201	0.028	1.6	0.7	Cell adhesion	Upregulated in spinal cord during EAE and suggested to be a regulator of oligodendrocyte differentiation. [54]
Protein Tyrosine Phosphatase, Receptor Type N	PTPRN	Q16849	− 0.49	− 2.201	0.028	1.6	0.6	Cell signalling	Important for proper secretion of hormones (insulin) and neurotransmitters [55]
Apolipoprotein H	APOH	P02749	− 0.32	− 1.992	0.046	1.7	0.8	Coagulation	May be associated with brain atrophy in healthy individuals [56]. Is the main antigen in antiphospholipid syndrome and may be associated with CNS related disease in these patients [57]
Fibrinogen gamma chain	FGG	P02679	− 0.61	− 2.201	0.028	1.5	0.5	Immune response, Acute phase protein	Important for proper T cell functioning and neutrophil pathogen clearance [37]. Regulator of microglia activation which may be important in pathogenesis of experimental autoimmune encephalomyelitis [58]
Alpha-1-B Glycoprotein	A1BG	P04217	− 0.39	− 2.201	0.028	2.6	0.7	Immune response, Acute phase protein	–
Beta-2-Microglobulin	B2M	P61769	− 0.44	− 1.992	0.046	1.7	0.8	Immune response, Adaptive immuntity	Increased in circulation in chronic fatigue syndrome [59] and identified as important in CSF of female chronic widespread pain patients [60]. CSF levels of B2M is suggested to reflect immune activation and lymphoid cell turnover in the CNS [61]
Complement C7	C7	P10643	− 0.48	− 2.201	0.028	2.1	0.5	Immune response, Innate immunity	–
Complement Factor B	CFB	P00751	− 0.38	− 1.992	0.046	1.7	0.6	Immune response, Innate immunity	Differentially expressed in AD CSF [62]
Complement C4B (Chido Blood Group)	C4B	P0C0L5	− 0.37	− 2.201	0.028	2.1	0.5	Immune response, Innate immunity	Differentially expressed in CSF of AD patients [62] and elevated in CSF of MS patients with active disease [63]
Hemopexin	HPX	P02790	− 0.33	− 1.992	0.046	1.7	0.7	Oxidative stress protection	Neuroprotective in stroke and intracerebral haemorrhages [64]. Increase in CSF following yeast-induced inflammation [65]

^†^Fold change is calculated as “(sample after infliximab − baseline sample)/baseline sample”. Proteins were identified in CSF of polyarthritis patients using label-free proteomics and uni- and multivariate data analysis

Based on the significant contribution to the separation in the PLS-DA model, significant alterations with infliximab treatment detected by univariate analysis and known associations to arthritis FGG, CADM3, HPX, CNTN1, A1BG, B2M and CFB were selected for closer studies and investigation of relations to clinical data.

**Fig. 2:**
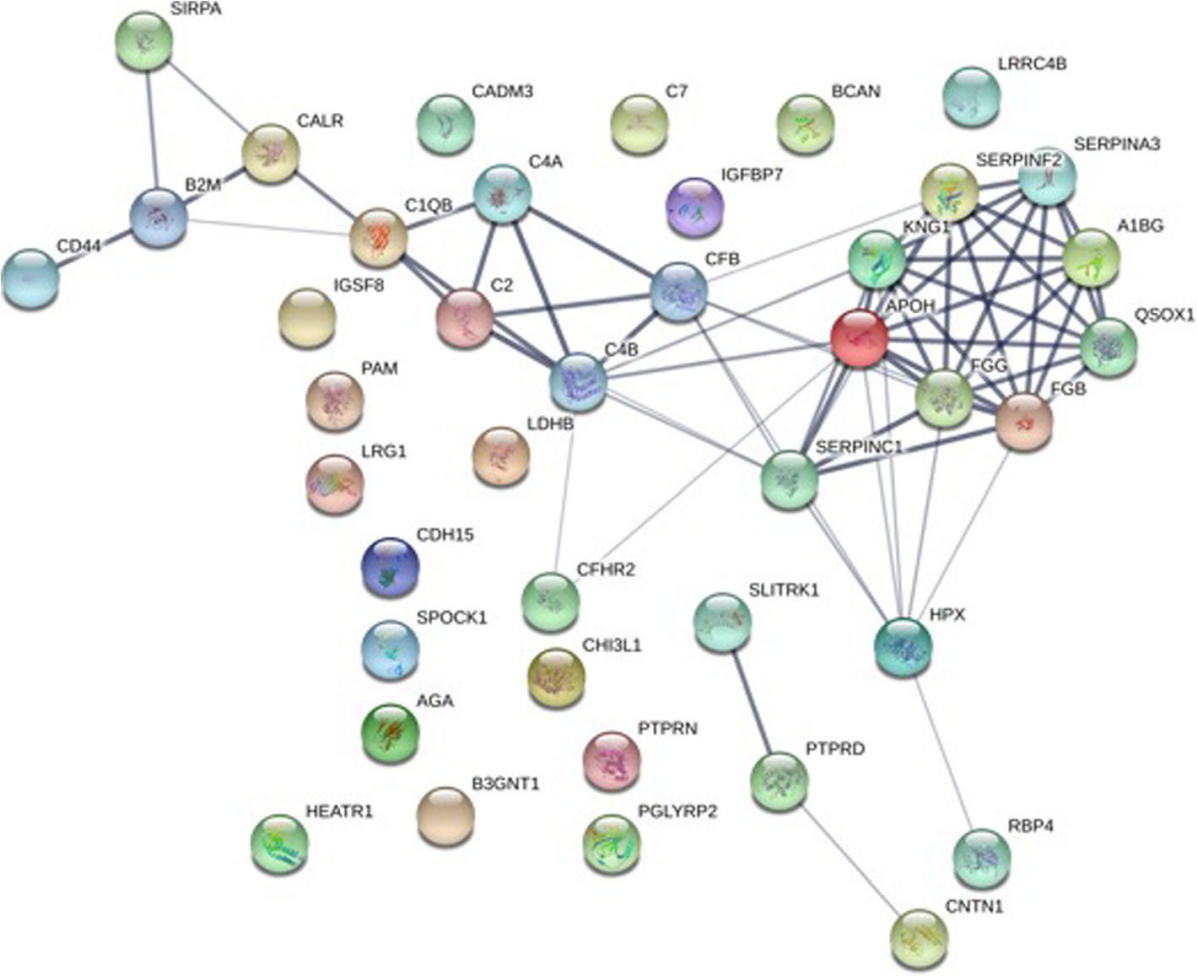
STRING (v.10.5)-based interaction analysis of the proteins identified by uni- and multivariate analysis as affected by infliximab treatment based on label-free proteomics data

Additionally, all proteins identified as affected by infliximab treatment by uni- and/or multivariate analysis from label-free proteomics data were analysed by the STRING online tool (v10.5) (Fig. 2) in order to reveal interactions among the identified proteins. Most interactions were described between proteins belonging to the complement and coagulation systems.

### Relative levels of CSF-proteins identified as regulated by infliximab treatment associate with systemic inflammation, function, pain and disease activity

**Fig. 3:**
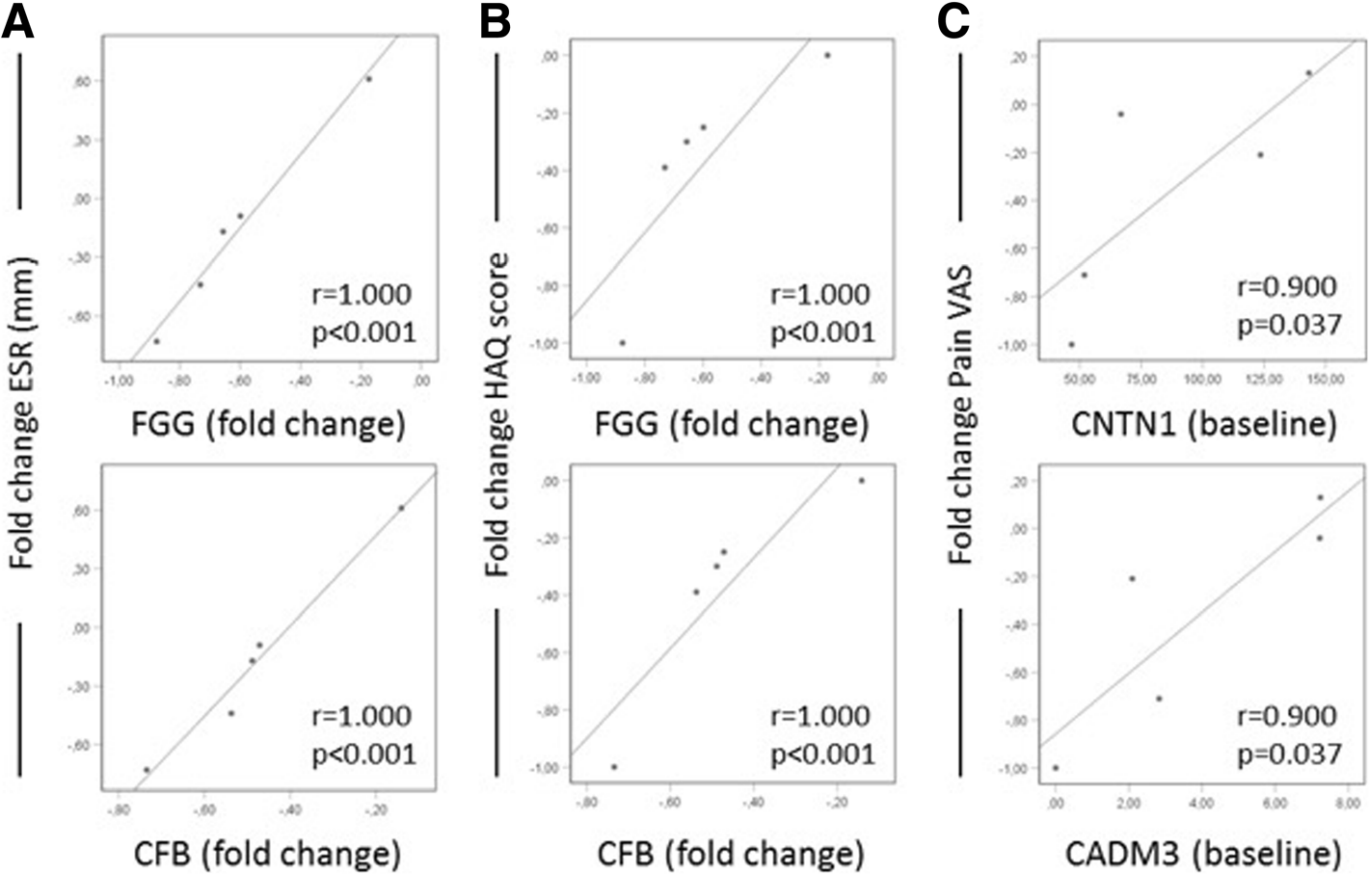
Spearman correlations between clinical measures and fold change or baseline NSAF values from label-free proteomic analysis of polyarthritis CSF samples at baseline and after 8 weeks of infliximab treatment (*n* = 5). **a** Fold change in ESR correlates positively to fold change in FGG and CFB. **b** Fold change in HAQ score correlates positively with fold change of FGG and CFB. **c** Fold change in VAS-pain correlates positively to NSAF values of CNTN1 and CADM3 at baseline. Fold change was calculated according to the formula “(samples following 8 weeks of imfliximab treatment − baseline samples)/baseline samples” and *p* < 0.05 was considered significant

When analysing the relations of identified candidate proteins to clinical measures, strong correlations were observed between the fold change of FGG and the fold change of ESR (*r*_s_ = 1.00, *p* < 0.001). Also, the fold change of CFB correlated to the fold change in ESR (*r*_s_ = 1.00, *p* < 0.001). Strong Spearman correlations were also observed between the fold change in both FGG and CFB and change in HAQ score during treatment (*r*_s_ = 1.00, *p* < 0.001; *r*_s_ = 1.00, *p* < 0.001, respectively). Additional correlations were also observed between both baseline CNTN1 and CADM3 and change in VAS-pain during treatment (*r*_s_ = 0.90, *p* = 0.037; *r*_s_ = 0.90, *p* = 0.037, respectively). Scatter plots are displayed in Fig. 3.

Taken together, our study shows that infliximab treatment not only affect systemic inflammation but also associate with changes in inflammatory markers in the CSF of arthritis patients, which may help explain the ability ofinfliximab and other TNF-blocking agents to relieve CNS-related symptoms.

## Discussion

The present study employed MS-based quantitative proteomics to identify CSF proteins that were affected during infliximab treatment. Initially, 31 proteins in CSF of the polyarthritis patients were found to be reduced with infliximab treatment. We then utilised multivariate analysis methods on all identified proteins (*n* = 306) applying stringent criteria to assess the proteins contributing the most to possible treatment-related differences, which included 11 of the 31 proteins affected by infliximab treatment (Additional file 1: Table S3). The reported functions of the majority of these 11 proteins are related to the immune system, including both innate and adaptive responses such as cell adhesion, complement, and coagulation systems. This is in line with TNF blockade being used as an anti-inflammatory treatment, and its peripheral anti-inflammatory effects are well documented in literature [8, 9, 14], and as shown in the present study, the levels of both ESR and CRP as well as disease activity scores were reduced after treatment.

Biologic macromolecules such as infliximab and TNFα do not readily pass the blood-brain barrier (BBB); however, there is a saturable receptor-assisted transport system for TNFα across the BBB [27]. Additionally, pro-inflammatory cytokines such as TNFα can alter BBB permeability through inflammatory damage to endothelial cells of the brain microvascular system [28]. The relative ratio of albumin between CSF and plasma is commonly used to evaluate BBB integrity [29, 30] and were reported normal for all patients throughout the present study. Interestingly, peptide sequences matching the infliximab antibody were identified in the tested polyarthritis CSF samples after infliximab treatment by the MS-based proteomics applied in this study (data not shown). One alternative route for passage of macromolecules entering the CNS could be at sites of modified BBB integrity, such as the circumventricular organs [31].

The monoclonal human-mouse chimeric antibody infliximab is one of the available biologic treatments binding TNFα. Although TNF blockade and other biologic drugs have been established as treatment strategy for more than a decade in chronic arthritis [10–13], much remain unknown regarding the effect of these drugs on central nervous mechanisms.

Spinal fluid samples are always very precious since the lumbar puncture procedure is rarely performed on arthritis patients. In this unique study, we have access to CSF from the same patients at two time points, before and after anti-TNF treatment enabling us to address these questions by mass spectrometry-based analysis. With this study, we are able to show that the effect of TNF-blockade is not limited to the synovium and systemic features, such as adhesion molecule regulation on endothelium [32], but also exerts effects in the CSF, which may help explain why this treatment strategy is able to ameliorate CNS-related symptoms to some extent. In support of this, animal studies of experimental arthritis have reported the ability of TNF blockade to exert substantial effects in the CNS, including a reduction of astrocyte activation and TNFα-dependent activation of stress-induced kinases that are both coupled to altered nociception [17, 18].

Our proteomics data is showing good overlap with data in cross-referenced studies (Additional file 2: Figure S2). As shown in Additional file 1: Table S4, all of the seven selected proteins identified as infliximab regulated by both uni- and multivariate analysis were found to be overlapping with proteins identified in at least one of the cross-referenced studies investigated. CNTN1, HPX and B2M were additionally reported as differentially expressed or regulated in CSF of the cross-referenced diseases. Taken together, this indicates their possible importance for disease features.

Fibrinogen gamma chain (FGG) was identified as significantly downregulated after infliximab treatment. This is an intriguing fact since FGG was also found to be decreased in arthritis plasma following TNF blockade (etanercept) [33] confirming the reasonability of our observation. Fibrinogen is involved in blood clotting, but has also been implicated as an inflammatory mediator in several diseases, including rheumatoid arthritis, multiple sclerosis (MS) and Alzheimer’s disease (AD) [34]. FGG has additionally been reported to be expressed at higher levels in rheumatoid arthritis synovial fluid [35], something that has also been shown for Alpha-1-B Glycoprotein (A1BG) [36], although little else is known about A1BG function. FGG showed strong association to both ESR levels and HAQ scores (Fig. 3) in our study where a more pronounced reduction of FGG after treatment corresponded to more pronounced reduction of ESR and HAQ reflecting the inflammatory nature of FGG and the beneficial effects of an overall reduced inflammatory burden in the patient.

Animal studies have shown that fibrinogen gamma can bind to the CD11b/CD18 complex often referred to as macrophage antigen complex-1 (Mac-1) [37], commonly expressed on immune cells including microglia [38]. Engagement of Mac-1 has been shown to modulate the inflammatory response of the Mac-1 expressing cell, although the exact nature of the modulation seems to depend on the cell type. In microglia, blocking of Mac-1 has been shown to inhibit H_2_O_2_ production in response to diesel exhaust particles, a known microglial inflammatory response inducer, thereby protecting against neuronal loss [39]. In Parkinson’s disease, Mac-1 expression on microglia has similarly been linked to prostaglandin E_2_ production, microgliosis, and subsequent neuronal loss [40]. In light of this, there may be a potential role of CSF-FGG in modulating microglial activation and inflammatory responses via interaction with Mac-1, which may affect CNS-related symptoms.

Regarding the other candidate proteins identified in this study, Contactin-1 (CNTN1) has been shown to be significantly upregulated after electric stimulation of the spinal cord in neuropathic pain patients [41]. CNTN1 is a protein involved in cell adhesion, important for formation of axon connections, and has been implicated in long-term depression [42]. Interestingly, also for cell adhesion molecule 3 (CADM3), another cell adhesion protein, relation to depressive symptoms has been reported [43]. In the present study, both proteins were reduced during infliximab treatment, suggesting that the regulation of these proteins is inflammation dependent. No association was found between the baseline levels of these proteins and inflammatory parameters; instead, an association with the magnitude of pain suppression was observed (Fig. 3). In line with this, animal studies have demonstrated that CNTN1 accumulates in axotomized dorsal root ganglion neurons, associates with the sodium channel Na(v)1.3 and may contribute to its hyper excitability involved in neuropathic pain signalling [44]. Taken together, we demonstrate that both CNTN1 and CADM3 may be involved in intrathecal processes regulating pain also in arthritis patients.

Hemopexin (HPX) is known to protect cells from oxidative stress which is frequently generated during an inflammatory response [45, 46]. Although HPX was significantly downregulated after infliximab treatment, no associations to clinical parameters were observed for this protein in our data. Changes in HPX level may be a consequence of the overall reduced inflammation in the treated patients, as it is also an acute phase protein.

The beta-2-microglobulin (B2M) protein is normally associated with the heavy chain of major histocompatibility complex, class I (MHC-I), which is responsible for antigen presentation on almost all nucleated cells [47]. However, during an inflammatory response, the circulating levels of free B2M protein are known to increase and increased CSF levels of B2M have been observed in painful disc degeneration [48]. In line with this, we observe increased baseline levels in CSF of our arthritis patients compared to controls, although this difference is not significant (data not shown), adding another indication of ongoing inflammation in the CSF of arthritis patients. B2M serum levels have also been reported to correlate with disease activity measures in rheumatoid arthritis [49]; however, no such correlation was found for CSF in the present study which might be due to our low number of study participants.

Complement factor B (CFB), also among the selected proteins for further investigation, was only identified in one of the cross-referenced studies. Identification of this protein in our study is still likely to be a true observation, although false positivity cannot be excluded. One explanation for its low number of overlapping observations could be that it is more specific to arthritis-related processes rather than to general inflammatory processes. This is the first study investigating the proteome in CSF of autoimmune arthritis wherefore overlap with other multiple studies may be absent for arthritis-specific proteins. CFB was found to be significantly reduced following infliximab and like FGG shows strong associations to reduction of ESR and HAQ score following treatment. CFB is a circulating complement factor involved in the complement cascade of the alternative pathway [50]. Inhibition of the alternative pathway by blocking CFB activity has been shown to be neuroprotective in an EAE mouse model [51]. TNFα have additionally been reported as an important regulator of CFB expression in macrophages [52] and human peripheral blood mononuclear cells (PBMCs) showing a dose-dependent CFB production upon TNFα treatment [53]. Considering these facts, it is then logical to attribute the reduced CFB levels observed in the CSF of our arthritis patients to the reduced TNFα levels induced by the infliximab treatment.

This investigation has several limitations that need to be taken into account when interpreting the results. The sample number is small, and the identified proteins should be recognised as potential markers in need for subsequent validation concerning significance in a general context. The fact that the included patients have different diseases and treatments may be of importance concerning differences in genetic predisposition and pathogenesis. On the other hand, the comparisons with only female patients and controls may decrease the possibility of heterogeneity in the results. Moreover, we included only patients with prednisolone doses below 10 mg. Strengths of the study include the unique investigation of CSF in polyarthritis patients and the potential to use serial sampling to investigate impact on CNS immune mechanisms and symptoms by a routinely prescribed biologic agent.

Taken together, we found that several proteins with inflammatory function were decreased in arthritis CSF after infliximab treatment, including proteins with additional neuro-immunomodulatory function such as FGG, CNTN1 and CADM3. We further show that in spite of the small and heterogeneous study cohort, several correlations between selected candidate proteins and clinical measures were observed.

## Conclusions

In conclusion, using proteomic profiling of CSF in polyarthritis, we have identified several intrathecal proteins with known inflammatory and/or neuro-immune function that were affected by TNF-blocking treatment. These results are in line with earlier findings of increased inflammatory mediators in CNS of both experimental and human arthritis [4] providing further evidence that the CNS is an important location to investigate in order to acquire a full understanding of the pathophysiology of arthritis and arthritis related symptoms.

## Additional files

Additional file 1: **Table S1.** Proteins significantly altered in CSF of polyarthritis patients after 8 weeks of infliximab treatment identified by unbiased label-free proteomic profiling and analysed by Wilcoxon signed rank test. **Table S2.** Full list of proteins detected in proteomic assay. Treatment effect was tested by Wilcoxon signed rank test for paired polyarthritis samples at baseline and after 8 weeks of infliximab treatment. *p* < 0.05 are marked in red. **Table S3.** Proteins detected by proteomic profiling contributing the most to the separation of arthritis CSF samples at baseline and after 8 weeks of infliximab treatment. **Table S4.** Proteins detected in proteomic profiling in CSF of polyarthritis patients overlapping with proteins detected in CSF in other diseases as well as proteins identified as differentially expressed or regulated in these diseases. (XLSX 58 kb)

Additional file 2: **Figure S1.** PLS-DA was performed on proteomic data. **Figure S2.** Overlap between proteins detected in CSF of polyarthritis patients at baseline and during infliximab treatment by proteomic profiling (polyarthritis (blue)) and proteins detected in CSF of healthy females (yellow), patients with multiple sclerosis (green) and patients with Alzheimer’s disease or mild cognitive impairment (red) in published studies. (DOCX 483 kb)

## Abbreviations

A1BG: Alpha-1B-glycoprotein
AD: Alzheimer’s disease
APOH: Apolipoprotein H
B2M: Beta-2-microglobulin
BBB: Blood-brain barrier
BL: Baseline
C4B: Complement C 4B
C7: Complement C7
CADM3: Cell adhesion molecule 3
CFB: Complement factor B
CNS: Central nervous system
CNTN1: Contactin-1
CRP: C-reactive protein
CSF: Cerebrospinal fluid
DAS28: Disease activity score 28
ESR: Erythrocyte sedimentation rate
FDR: False discovery rate
FGG: Fibrinogen gamma chain
HAQ: Health assessment questionnaire
HPX: Hemopexin
IFX: Infliximab
IGFBP7: Insulin-like Growth Factor Binding Protein 7
IL-1β: Interleukin-1beta
IL-6: Interleukin-6
LC: Liquid chromatography
Mac-1: Macrophage antigen complex-1
MS: Mass spectrometry
MS: Multiple sclerosis
NINDC: Non-inflammatory neurological disease control
NSAF: Normalised spectral abundance factor
ON: Overnight
PBMCs: Peripheral mononuclear cells
PCA: Principal component analysis
PLS-DA: Partial least squares discriminant analysis
PTPRN: Protein Tyrosine Phosphatase Receptor Type N
RA: Rheumatoid arthritis
TCEP: Tris(2-carboxyethyl) phosphine hydrochloride
TEAB: Triethyl ammonium bicarbonate
TMT: Tandem mass tag
TNFα: Tumour necrosis factor alpha
VAS: Visual analogue scale
VIP: Variable influence in projection
