# Supplementary material for: Mass spectrometry-based analysis of cerebrospinal fluid from arthritis patients—immune-related candidate proteins affected by TNF blocking treatment
Source: Arthritis Res Ther. 2019 Feb 15;21:60. doi: 10.1186/s13075-019-1846-6 (PMC6377734; doi:10.1186/s13075-019-1846-6)
Supplement: Supplementary file 2 — Figure S1. PLS-DA was performed on proteomic data. Figure S2. Overlap between proteins detected in CSF of polyarthritis patients at baseline and during infliximab treatment by proteomic profiling (polyarthritis (blue)) and proteins detected in CSF of healthy females (yellow), patients with multiple sclerosis (green) and patients with Alzheimer’s disease or mild cognitive impairment (red) in published studies. (DOCX 483 kb) [file 13075_2019_1846_MOESM2_ESM.docx]

**Mass spectrometry-based analysis of cerebrospinal fluid from arthritis patients – immune related candidate proteins affected by TNF blocking treatment**

Johanna Estelius^1^, Johan Lengqvist^1^, Elena Ossipova^1^, Helena Idborg^1^, Erwan Le Maître^1^, Magnus LA Andersson^2^, Lou Brundin^2^, Mohsen Khademi^2^, Elisabet Svenungsson^1^, Per-Johan Jakobsson^1^, Jon Lampa^1^

**Supplementary Figure 1**

**
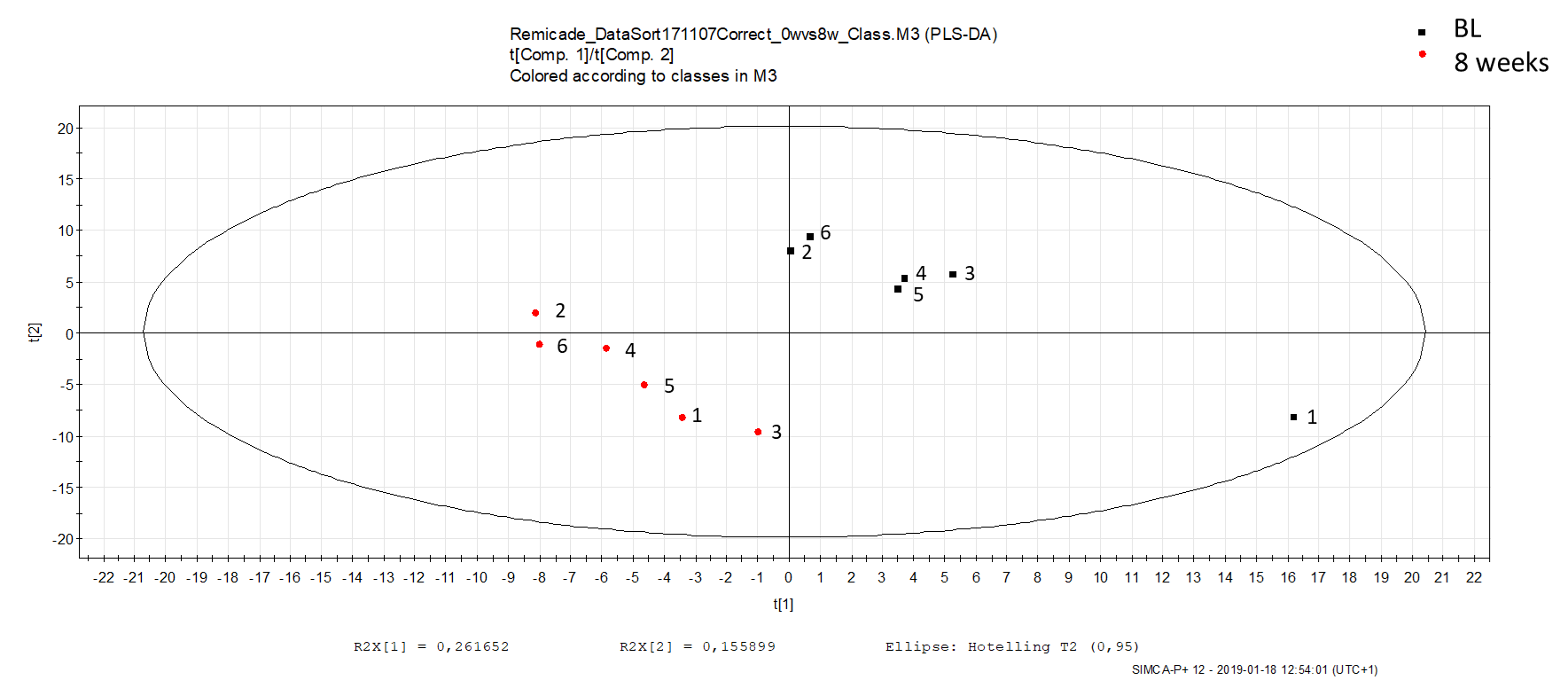
**

**Fig. S1** PLS-DA was performed on proteomic data

1. Scores plot obtained from PLS-DA on label-free MS proteomic data comparing baseline and 8w. Samples before and after treatment could be separated. The PLS-DA model is not significant but was used so select proteins for further investigation.
2. Corresponding loadings plot (PLS weights) was obtained. Labels =protein names

**Supplementary figure 2**

**
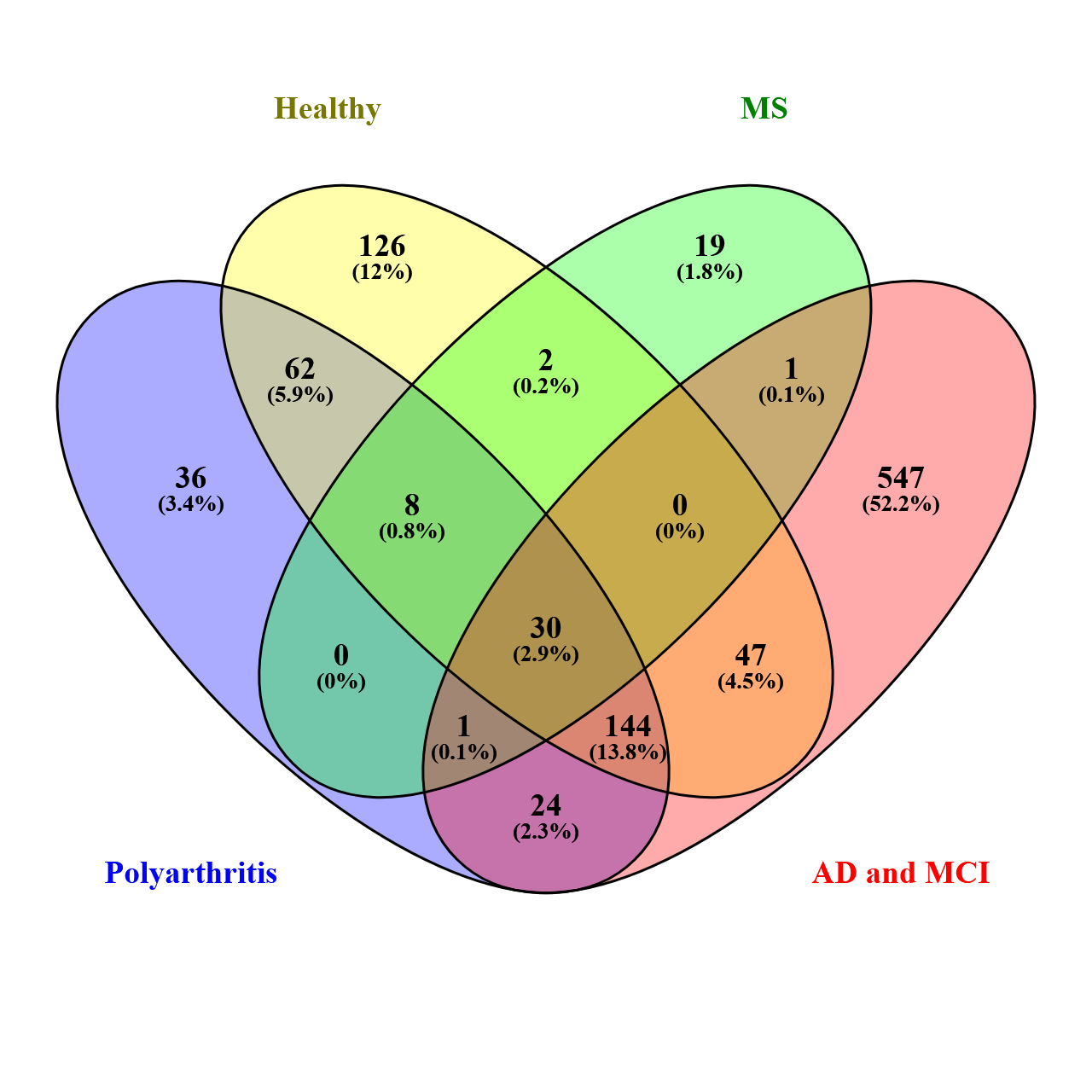
**

**Fig. S2** Overlap between proteins detected in CSF of polyarthritis patients at baseline and during infliximab treatment by proteomic profiling(polyarthritis (blue)) and proteins detected in CSF of healthy females (yellow), patients with multiple sclerosis (green) and patients with Alzheimer’s disease or mild cognitive impairment (red) in published studies.

**References**

1. Percy AJ, Yang JC, Chambers AG, et al. Multiplexed MRM with Internal Standards for Cerebrospinal Fluid Candidate Protein Biomarker Quantitation. J Proteome Res 2014;**13**(8):3733-47.
